# Regulation of gingival fibroblast phenotype by periodontal ligament cells in vitro

**DOI:** 10.1111/jre.12971

**Published:** 2022-01-17

**Authors:** Devy F. Garna, Francis J. Hughes, Mandeep S. Ghuman

**Affiliations:** ^1^ Centre for Host‐Microbiome Interactions Faculty of Dentistry Oral & Craniofacial Sciences King's College London London UK; ^2^ Department of Periodontology Faculty of Dentistry Padjadjaran University Bandung Indonesia

**Keywords:** fibroblast, gingiva, periodontal ligament, stem cell

## Abstract

**Objectives:**

Stem cell transplantation has shown modest effects on periodontal tissue regeneration, and it is still unclear how regenerative effects utilizing this modality are mediated. A greater understanding of the basic interactions between implanted and host cells is needed to improve future strategies. The aims of this study were to investigate the effects of periodontal ligament (PDL) cells on expression of periodontal markers and alkaline phosphatase (ALP) activity of gingival fibroblasts (GF).

**Materials and Methods:**

Primary human PDL cells were co‐cultured with primary GF cultures either by direct co‐culture with subsequent FACS sorting or indirect co‐culture using transwell cultures and PDL cell conditioned medium. Expression of periodontal markers, asporin, nestin, and periostin, was assessed by qPCR and immunofluorescence staining. Alkaline phosphatase (ALP) expression was assessed by qPCR, histochemical staining, and activity assessed by para‐nitrophenol enzymatic assay. Single cultures of PDL cells and GF were used as controls. The role of Wnt signaling on ALP activity was assessed via Dkk1‐mediated inhibition.

**Results:**

PDL cells significantly upregulated expression of PDL markers in GF with both direct and indirect co‐culture methods when compared to controls (6.05 vs. 0.73 and 59.48 vs. 17.55 fold change of asporin expression). PDL/GF cell co‐cultures significantly increased ALP activity in GF when compared with single GF cultures. Similar results were obtained when using conditioned medium isolated from PDL cell cultures. Dkk1 caused dose‐dependent reduction in ALP activity of GF cultured in PDL cell conditioned medium.

**Conclusions:**

PDL cells stimulate expression of periodontal markers and osteogenic capacity of gingival fibroblasts via paracrine signaling which can be partially inhibited with addition of the Wnt antagonist, Dkk1.Further studies are required to identify specific secreted factors responsible for this activity.

## 1 INTRODUCTION

There is considerable interest in the possibility of using mesenchymal stem cell (MSC) therapies to promote periodontal regeneration.^1^ A number of animal studies have now demonstrated the principle that implantation of either autologous or allogeneic MSC derived from different sources may contribute to the outcome of periodontal regeneration in preclinical models,^2^ but this has far only translated modestly in human studies.^3^

The presence of high numbers of MSCs within the PDL, as shown by their differentiation potentials, self‐renewal, and ability to contribute to periodontal tissue regeneration when implanted into periodontal defects has been formally demonstrated^4^ and with greater consistency compared to those derived from other sources.^5^ It is also known that in addition to the potential for exogenously applied stem cells to contribute directly to tissue regeneration, they also have properties of immunomodulation, homing, and recruitment of endogenous host cells via paracrine signaling mechanisms.^2^ In preclinical models, there is some evidence that periodontal ligament‐derived MSC (PDLSC) may contribute directly to new cementum formation,^4^ but also that they might stimulate periodontal regeneration indirectly through their interaction with existing endogenous host cells^6^, ^7^

Periodontal ligament cells have distinct phenotypic features including the expression of alkaline phosphatase, nestin, asporin, and periostin.^8^, ^9^, ^10^, ^11^ Asporin (periodontal ligament associated protein 1, PLAP‐1) and periostin are matrix proteins that are particularly highly expressed within the periodontal ligament. Nestin is a neural crest‐derived cell marker, and its expression in PDL cells reflects their embryological origin. Alkaline phosphatase is also constitutively expressed in the PDL, and its expression is upregulated during osteoblastic differentiation.

A number of studies have demonstrated the presence of MSCs in both healthy and inflamed gingivae,^12^, ^13^ which raise the issue of whether these cells might also contribute to periodontal tissue regeneration and might be recruited during regenerative wound healing to contribute to the healing tissues.^14^, ^15^, ^16^

Therefore, the aim of study described here was to investigate the hypothesis that PDLSC may induce the expression of periodontal markers in gingival fibroblasts in vitro.

## 2 MATERIALS AND METHODS

### 2.1 Overview of experiments

To investigate the interactions between PDL and gingival cells, firstly PDL and gingival cells were separately labeled with cell tracker fluorescent labels and then co‐cultured. PDL and gingival cells were then sorted by flow cytometry and expression of PDL markers determined by qPCR. Further co‐culture experiments were then carried out using transwell plates to physically separate PDL and GF cells in the same culture medium. Finally, further experiments were carried out using conditioned medium collected from PDL and GF cells. Preliminary investigation of signaling pathways involved in cell interactions was then carried out by adding a Wnt signaling inhibitor, Dkk1, and a BMP signaling inhibitor, noggin, to GF cultures treated with PDL conditioned medium, and measuring effects on ALP expression.

### 2.2 Isolation and characterization of cells

Explant cultures of gingival fibroblasts were established from tissues from healthy third molar teeth extracted from the patients aged between 20 and 35 years old, with no significant medical problems and no smoking history. All patients were attending a surgical appointment at the Department of Oral Surgery, Guy's Hospital. Kent NHS Research Ethics Committee approved the protocol (Reference No: 11/LO/0259), and informed written consent was obtained from all the subjects. Detailed methods of cell isolation are described in additional Supplemental Methods. Three gingival cell lines were derived in this way (designated GF1–GF3). In addition, a further primary human gingival fibroblast line (GF4) was purchased from ScienCell (Caltag Medical Systems, Buckingham UK, Catalog no #2620). These cells were obtained at passage 1 and cultured in fibroblast medium (ScienCell cat no 2301) until reaching the same passage as other primary GF. The fibroblast medium was replaced one day before the experiments were started. Experiments were carried out on cells at passage 4–7. Periodontal ligament stem cell cultures were obtained from 3 different donors. Isolation and characterization of these PDL stem cell cultures was carried out by our group and has been previously described.^17^ All PDL cell lines used in the experiments were derived from individuals with similar demographic characteristics as those for gingival cells. PDL cells expressed a high proportion of MSC markers CD44, CD146, CD105, and CD90 (ranging from 86%‐99% in flow cytometry analysis) and a very low level of the hematopoietic cell markers CD45 and CD34 (<1%). Also, these PDL cell lines were able to differentiate into three lineages in vitro (osteoblastic, adipocytic, and chondroblastic lineage) when cultured in appropriate conditions.

To characterize the GF cell lines, cells were tested for the expression of the same cell surface markers including CD44, CD146, CD105, CD90, CD45, and CD34 by flow cytometry. Cell differentiation potentials were investigated by culturing cells in osteogenic, adipogenic, and chondrocytic media for up to 28 days and stained with alizarin red, oil‐red‐O, and alcian blue, respectively, for osteoblast, adipocyte, and chondroblastic differentiation. Detailed methods of these experiments are described in Supplemental Methods. To investigate expression of the putative periodontal markers asporin, periostin, nestin, and alkaline phosphatase, gene expression was investigated by qRT‐PCR.

For co‐culture experiments and those with conditioned media, experiments were carried out in triplicate and repeated three times using PDL cells from the three different cell lines. Data from separate experiments were pooled by calculation of the means from each test.

### 2.3 qRT‐PCR

Total RNA was extracted using TRIzol reagent (Ambion, Warrington, UK). Cells in 12‐well plates were lysed in 600ml TRIzol reagent and transferred into Phase Lock Gel Heavy tubes (5 prime, VWR, Leicestershire, UK) according to the manufacturer's instructions. RNA purity and quantity were assessed by Nano Drop 1000 Mass spectrometer (Fisher Scientific). Ratios of A260/A280 between 1.8 and 2 were considered to be of high purity and used for cDNA synthesis. Possible contaminating DNA was removed and cDNA prepared from 1 μg RNA using QuantiTect Reverse Transcription Kit (Qiagen, West Sussex, UK) according to the manufacturer's instructions. qRT‐PCR was performed with a Rotor‐Gene 6000 thermal cycler (Qiagen) using Brilliant III Ultra‐Fast SYBR Green qPCR Master mix (Stratagene, Agilent Technologies, Cheshire, UK) and primer pairs as listed in the Table 1. PCR conditions consisted of 1 cycle of 95°C for 3 min. and 40 cycles of 95°C for 10 s. and 60°C for 10 s. followed by melting analysis of 1 cycle with gradual increase from 65 to 95°C. RPL13a was used as the housekeeping gene.

**TABLE 1:** List of primers and sequences for Sybr green based RT‐qPCR assays

Gene	Gene Acc	Forward 5’−3’	Reverse 5’−3’
RPL13a	NM_000977	CATCGTGGCTAAACAGGTACTG	GCACGACCTTGAGGGCAGCC
Asporin (ASPN)	SY_130414037	CCCTTCAGGATTACCAGAGTTG	TTGGCACTGTTGGACAGAAG
Periostin (POSTN)	SY_130414039	TGACACAACCTGGAGACTGG	GAGCATTTTTGTCCCGTATCA
Nestin (NES)	SY_140307726	CTCCAAGACTTCCCTCAGCT	TCAGGACTGGGAGCAAAGAT
Alkaline Phosphatase (ALP)	NM_000478	AACACCACCCAGGGGAAC	TGGCTGGTTCACTCTCGT

### 2.4 Analysis of qRT‐PCR data

To calculate relative expression, all samples were normalized housekeeping gene expression (RPL13a) cycle threshold (Ct) value and then normalized to the average of PDL Ct value at baseline day 0 (24 h after seeding the cells). Fold changes of expression of gene of interest were assessed using the comparative 2^−(delta delta Ct)^ method. Data are expressed as means ± *SD*.

### 2.5 Direct co‐cultures

PDL and GF cells were cultured and grown in normal media at 37°C. After the cells reached confluence, the cells were subcultured with trypsin/EDTA and stained with cell tracker fluorescent markers. CellTracker^TM^ Green (C7025 Invitrogen) and CellTracker^TM^ Orange (C34551 Invitrogen) were used to label gingival fibroblasts and PDL cells, respectively, in direct co‐culture and their single culture controls. When the cells were approximately 90% confluent, the cells were detached with trypsin after washing with PBS and centrifuged for 5 min at 1100 rpm. Then, the pre‐warmed 5 μM CellTracker^TM^ dye working solution was added and incubated for 30 min in CO_2_ incubator. The cells were centrifuged and washed by PBS to remove excess dye. After being stained with cell trackers, the cells were divided into two groups, those for FACS sorting and those not for FACS.

PDL cells and GF were directly co‐cultured in the same wells of 12‐well plates with the ratio of PDL cells to GF of 1:3. As a control group, single cultures of PDL cells and GF were used and seeded at the same amount as the total cell amount of co‐culture. Initial density of PDL cells in co‐culture was 5 × 10^3^ cells/well, whereas GF was 1.5 × 10^4^ cells/well. Meanwhile for each single culture of PDL cells or GF, 2 × 10^4^ cells/well was plated. For subsequent gene expression studies, as additional controls, comparing sorted with non‐sorted cells tested the effect of cell sorting on gene expression.

Fluorescence activated cell sorting (FACS) of co‐cultured PDL cells and GF was conducted after 3 days of culture in normal media. The BD FACSAria II was used for cell sorting according to different cell fluorescence labeling. The standard flow cytometry Electrostatic Sorting was used for cell sorting into designated containers. BD FACSDiva software was used for analysis. CellTracker^TM^ Green was the FITC channel at 488 nm excitation and 530/30 collections. CellTracker^TM^ Orange was the PE channel at 488 nm excitation and 575/26 collections.

Single cultures with cell tracker staining were used as positive controls, and unstained cells were used to set optimal side and forward scatter voltages on the FACS machine and prevent unwanted cells before cell sorting. Cells were sorted on a 100‐μm nozzle. All sorted cells were cultured in new 12‐well plates as a single culture and after 24 h, the cells were processed for total RNA extraction and real‐time PCR. Cells were then cultured until reaching 80% confluence and lysed with TRIzol^®^ Reagent (Thermo Fisher, Scientific) for processing of total RNA extraction.

### 2.6 Indirect co‐cultures

Indirect co‐cultures were established using transwell cell culture inserts (Corning Costar, Cambridge, MA) with 0.4‐μm pore‐size filter membranes. GF (4.5 × 10^4^ cells/ml) were grown on the transwell inserts on top, whereas PDL cells were seeded in the lower layer at a ratio of 1:3. As controls, GF cells were seeded into both top and bottom compartments of the transwell plates and likewise control cultures were also obtained with PDL cells in top and bottom compartments. Growth medium was added to cover both cell layers and was cultured for 3 days before proceeding with RNA extraction.

### 2.7 Conditioned medium

PDL cells and GF were grown with normal media in flask T75 with the same initial density of 1 × 10^5^ cells at 37°C in a humidified 5% CO_2_, 95% air atmosphere. After the cells reached 90–95% confluence, the normal media were removed and the cells were washed with PBS 3×; then, 5 ml of normal medium without serum was added into the flask. The cells were incubated at 37°C in a humidified 5% CO_2_, 95% air atmosphere for 24 h. Conditioned medium was collected and filtered with 0.22μm strainer and stored frozen at −20°C prior to use in experiments.

PDL and GF cells were seeded at a density 15 000 cells per wells into 12‐well plates in triplicates at passage 5. All the cells were grown with normal media (NM), and after reaching 90–95% confluence, medium was removed, the cells were washed with PBS and the collected conditioned medium was supplemented with the addition of 1% serum and then cultured for 3 days before processing to RNA extraction. Additional controls of normal medium supplemented with the addition of 1% serum were also employed.

### 2.8 Alkaline phosphatase (ALP) assays

Cell culture medium was removed, and cells were washed with 100 μl PBS. After washing, PBS was discarded and 50 μl distilled water was added into the wells. Cell plates were placed in the incubator at 37°C for 15 min and transferred into −80°C for 20 min. This freeze‐thaw process was repeated three times.

A serial dilution of p‐nitrophenol standard (Sigma‐Aldrich) was prepared down to 3.125 μg/ml with the final volume 100 μl. The substrate reagent was prepared fresh for each run and made from 20 mg of p‐Nitrophenyl phosphate, 17 mg of magnesium chloride hexahydrate, 40 μl of Triton X‐100 and 5 ml 0.1 M Glycine, pH 10.3. 50 μl of the substrate reagent (all Sigma‐Aldrich) was added to all the samples. The plates were covered with aluminum foil and shaken for 2 min on a Titertek plate shaker. The absorbance was read immediately at 410 nm (reference wavelength 630 nm) on the Dynatech reader or 405 nm on the Biorad reader. The reading process was repeated after the plates had been incubating for 20 min. A standard curve of known concentrations of PNP was constructed for each assay and used to convert OD readings to ALP activity expressed in arbitrary units of PNP concentration.

Histochemical staining for ALP was carried out using the azo‐dye coupling method.^18^ Cells were fixed in 4% formaldehyde. Substrate solution was prepared by dissolving 60 mg napthol AS phosphate (Sigma‐Aldrich) in 1 ml dimethyl formamide (Sigma‐Aldrich) and added to 200 ml of 0.2 M Tris HCl buffer (pH 9.0). 20mg Fast Blue BB (Sigma‐Aldrich) was added to 50 ml substrate solution, and the cells were incubated with 3 ml/well filtered substrate solution for 1 h.

### 2.9 Effects of inhibition of Wnt signaling on ALP expression

In preliminary experiments to characterize the nature of the signaling molecules present in conditioned media, we added the Wnt inhibitor Dkk‐1 (R&D Systems) to conditioned media at concentrations from 0 to 1000 ng/ml prior to treating GF. The cells were then cultured for 3 days. After 3 days, ALP activity was assessed and results were normalized to results obtained with medium without inhibitor.

### 2.10 Statistical analysis

All the experiments were carried out in triplicate, and one‐way ANOVA with Tukey or Bonferroni post‐hoc test was performed for statistical analysis using GraphPad Prism version 5.00 (GraphPad Software, San Diego California). A *p*‐value of <.05 was considered statistically significant.

## 3 RESULTS

**FIGURE 1:**
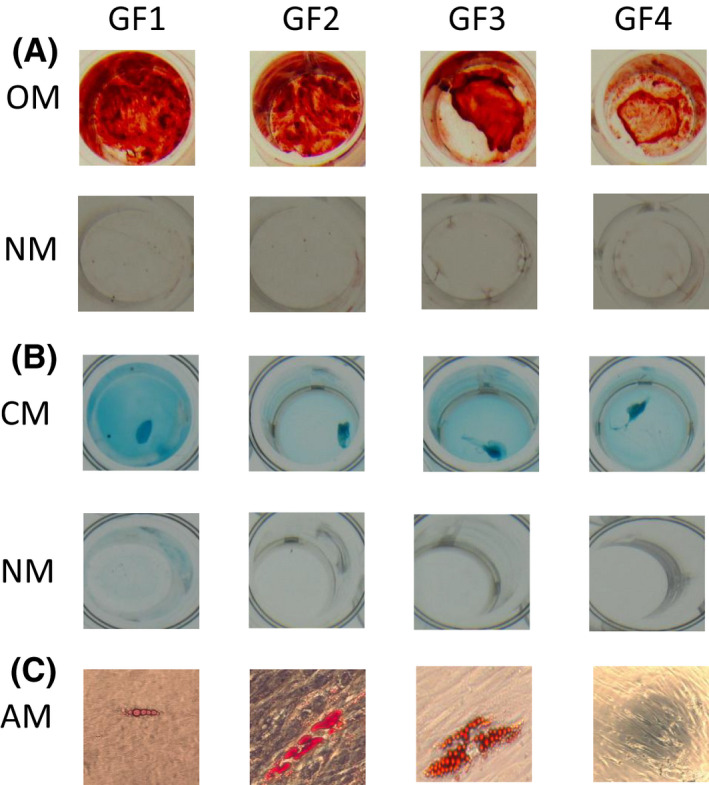
Staining of gingival fibroblasts following induction for 21 days in differentiation medium. Representative photographs (from 3 independent experiments) of 4 independent primary gingival fibroblast lines (GF1‐4) undergoing osteogenic (OM), adipogenic (AM), and chondrogenic (CM) differentiation following induction in differentiation medium for 21 days. Control cultures used normal medium. The ability for lineage‐specific differentiation was assessed for osteogenesis by staining with alizarin red for calcium deposition (A), for chondrogenesis by staining with alcian blue (B), and for adipogenesis by staining with Oil Red O for lipid accumulation (magnification, ×200) (C)

A very high percentage of cells in gingival fibroblast cultures 1, 2, and 3 (GF 1–3) expressed the mesenchymal stem cell surface markers CD44, CD90, and CD146 and very low levels of CD34 and CD45 (Table 2). Cells also expressed CD105, although in GF‐1 and GF‐2 lines this was lower than in GF‐3 (approx. 65% vs. 97%). In contrast, the GF‐4 cell line showed a lower proportion of cells expressing these markers, particularly CD146 and CD105. GF1‐3 lines also showed osteoblastic, adipocytic, and chondrogenic differentiation when cultured in inductive media for 21 days, whereas GF‐4 showed a lesser extent, particularly osteogenic and adipocytic (Figure 1).

**TABLE 2:** Percentage of gingival fibroblast (GF) cells expressing MSC cell surface markers (mean ± *SD*)

	CD 146	CD 105	CD 44	CD 90	CD 34	CD 45
GF 1	93.07 ± 4.10	63.96 ± 48.42	99.85 ± 0.07	98.17 ± 1.72	0.43 ± 0.45	0.37 ± 0.46
GF 2	83.93 ± 16.05	67.07 ± 8.71	99.85 ± 0.07	99.36 ± 0.56	0.78 ± 0.63	0.69 ± 1.13
GF 3	89.67 ± 9.47	97 ± 3.54	99.95 ± 0.07	95.13 ± 8.17	0.92 ± 0.95	0.08 ± 0.1
GF 4	7.78 ± 13.18	1.54 ± 2.39	85.10 ± 14.35	79.73 ± 7.43	0.37 ± 0.63	0.6 ± 0.22

All GF lines showed varying levels of constitutive expression of periostin, asporin, nestin, and alkaline phosphatase, with GF‐1 showing the highest expression of periostin, GF‐2 of asporin, and GF‐3 and GF‐4 of nestin and alkaline phosphatase (Figures S1 and S2). On the basis of all these results, GF‐3 was selected and used for all subsequent experiments except where indicated.

### 3.1 Direct co‐culture with FACS sorting

**FIGURE 2:**
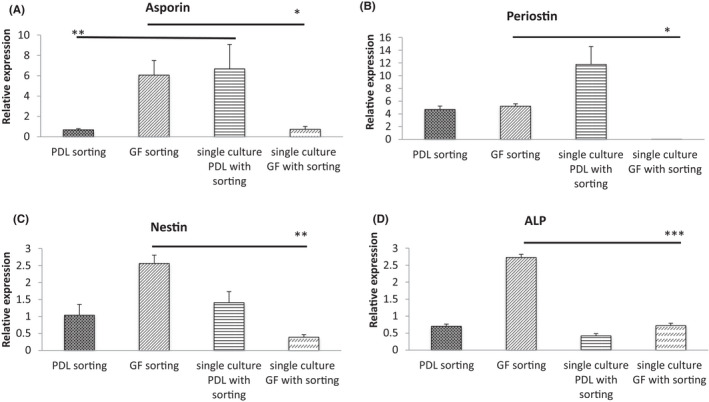
Changes in PDL cell marker gene expression in directly co‐cultured gingival fibroblasts and periodontal ligament cells following FACS. Gingival fibroblasts (GF) and periodontal ligament cells (PDL) were co‐cultured directly for 3 days with a GF:PDL ratio of 3:1. Marker expression levels were assessed by qPCR and expression normalized to RPL13a expression following FACS. Data presented as mean ± *SD* from 3 experiments. One‐way ANOVA with Bonferroni's post‐test was used for statistical analysis. *p < .05; **p < .01; ***p < .001. Only statistically significant differences indicated

Following co‐culture for 3 days and subsequent re‐sorting into either GF or PDL cells, there was a marked upregulation of all marker genes tested in GF cells when compared to GF monoculture controls (Figure 2). FACS gating, stained co‐cultures, and immunofluorescent detection of asporin, periostin and nestin are shown in Figure S3. Following co‐culture, PDL cells showed down‐regulation of asporin. In addition, it was clear that the process of FACS sorting itself markedly decreased the overall gene expression for all markers tested (eg, asporin expression in GF increased 5.77 fold without sorting compared to 0.73 fold after sorting) (Figure S4).

### 3.2 Periodontal marker expression in indirect co‐cultures and conditioned medium

**FIGURE 3:**
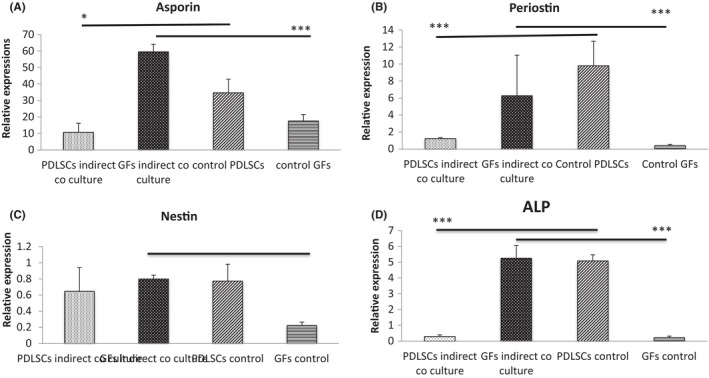
Gene expression of (A) asporin; (B) periostin; (C) nestin; and (D) alkaline phosphatase following indirect co‐culture of periodontal cells (PDLSC) and gingival fibroblasts (GFs). PDLSC and GF were grown transwell co‐cultures in normal media for 3 days. Single cultures either of PDLSC or GFs acted as control group. Marker expression levels were assessed by qPCR and expression normalized to RPL13a expression. Data represented as mean ± *SD*. Data were generated from using 3 independent primary PDLSCs lines and 1 independent primary GF line. Three replicates were tested for each cell line. One‐way ANOVA with Bonferroni's post‐test was conducted for statistical analysis. * *p *< .05; *** *p *< .001. Only statistically significant differences indicated

In order to determine whether interaction between the cell types was independent of the need for cell‐cell contact, we used transwell cultures which physically separated the GF and PDL cells in the same culture. GF cells that were grown with PDL cells in transwell co‐cultures showed marked upregulation of all markers tested. PDL cells showed marked down‐regulation of alkaline phosphatase, periostin, and asporin when grown in the presence of GF cells (Figure 3).

**FIGURE 4:**
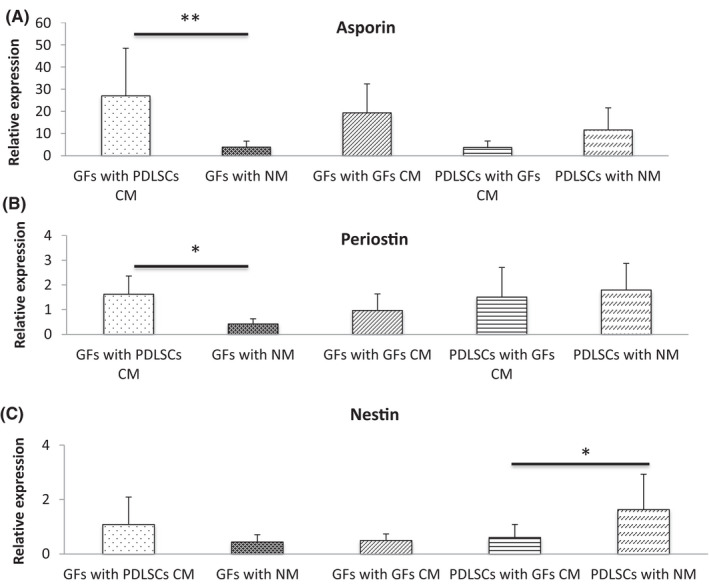
Gene expression of (A) asporin; (B) periostin; and (C) nestin following culture of periodontal cells (PDLSC) and gingival fibroblasts (GFs) in conditioned media (CM). GFs were cultured with conditioned medium (CM) from PDLSC and GF CM, whereas PDLs were cultured with GF CM for 3 days. As a control group, samples were cultured in normal media (NM). Marker expression levels were assessed by qPCR and expression normalized to RPL13a expression. Data represented as mean ± *SD*. Data were generated from using 3 independent primary PDLSCs lines and 1 independent primary GF line. Three replicates were tested for each cell line. One‐way ANOVA with Bonferroni's post‐test was used for statistical analysis. * *p *< .05; ** *p *< .01. Only statistically significant differences indicated

To follow on from the results of indirect co‐culture experiments, cells were treated with conditioned medium (CM) from PDL and GF cultures. There were no significant differences between the effect of GF CM and PDL CM on expression of the tested periodontal marker in GF cells (Figure 4). However, GF cultured in PDL CM induced a significantly greater upregulation of asporin and periostin expression compared with GFs grown in normal medium. GF CM had no significant effects on marker expression on GF when compared with normal medium, but significantly reduced expression of nestin only in PDL cell cultures.

**FIGURE 5:**
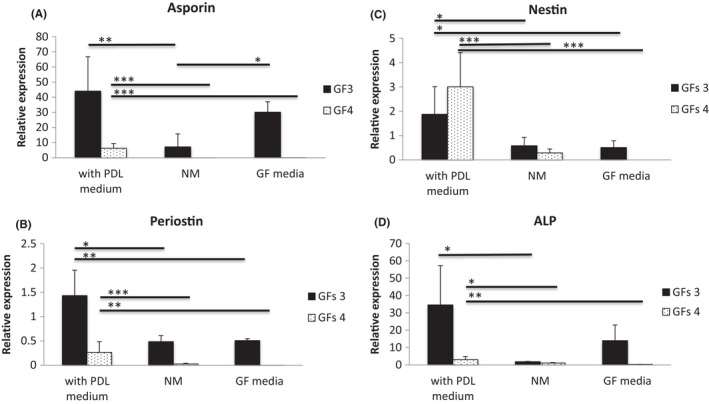
Comparison of (A) asporin; (B) periostin; (C) nestin; and (D) alkaline phosphatase gene expression in 2 primary gingival fibroblast (GF) lines cultured in condition medium. GFs were cultured with conditioned medium (CM) from PDLSC and GF CM for 3 days. As a control group, samples were cultured in normal media (NM). Marker expression levels were assessed by qPCR and expression normalized to RPL13a expression. Data represented as mean ± *SD*. Three replicates were tested for each cell line. One‐way ANOVA with Bonferroni's post‐test was used for statistical analysis. * *p *< .05; ** *p *< .01; ***p < .001. Only statistically significant differences indicated

These experiments were repeated with a second cell line (GF‐4) and compared head to head for periodontal markers (Figure 5A‐C) and ALP expression (Figure 5D) with GF‐3, notable for having the highest percentage of CD105 (Table 2) and both ALP expression and activity levels (Figure S2). In contrast, GF‐4 had shown marked phenotypic differences from the other cell lines that had been initially characterized. As with the previous experiment, GF‐3 showed a similar trend to the previous experiments in elevated expression of markers when cultured in PDL CM. Compared to GF‐3, used in all the experiments previously, GF‐4 showed lower expression levels of all markers tested when cultured in PDL and GF CM, except for nestin in PDL CM cultures (Figure 5). Expression of all markers and ALP was significantly more upregulated in GF‐4 cultured in PDL CM when compared with GF CM (Figure 5). Similarly nestin and periostin were also significantly more upregulated in GF‐3 PDL CM cultures when compared with those in GF CM. Furthermore, apart from asporin in GF‐3, expression of the markers and ALP was not significantly different between GF CM and normal media cultures.

### 3.3 Alkaline phosphatase activity

ALP protein activity was tested using both co‐culture and conditioned medium methods by measuring enzyme activity and histochemical staining.

**FIGURE 6:**
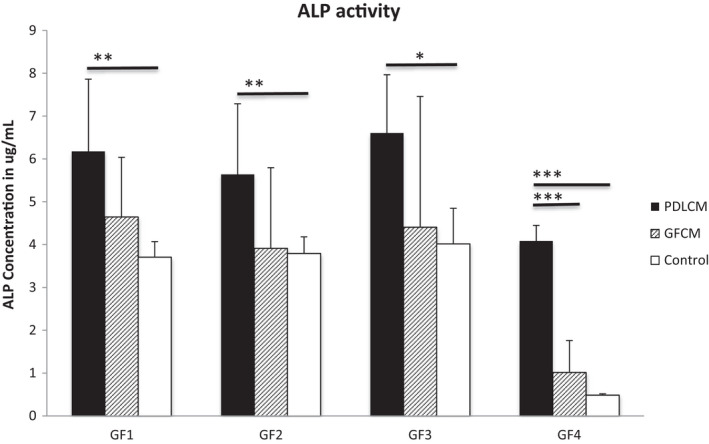
ALP activity of 4 independent primary gingival fibroblast (GF) lines cultured in PDL condition medium (PDL CM). GF were cultured in PDL CM for the test group for 3 days. As a control group, samples were cultured in normal media. All the samples carried out in triplicates and repeated twice. Data shown as mean ± *SD*. Three replicates were tested for each cell line. One‐way ANOVA with Bonferroni's post‐test was used for statistical analysis. *** *p* < .001. Only statistically significant differences indicated

Histochemical staining was used to test ALP production in direct co‐cultures compared to monotype cultures, and the intensity of alkaline phosphatase staining was quantified by plate reader. Co‐cultures treated with osteogenic medium showed a significant increase in alkaline phosphatase activity compared to GF single cultures (Figure S5). Although enzymatic activity assessment of para‐nitrophenol (PNP) release showed that PDL CM consistently elevated ALP activity in GF1‐3 cell lines above that of GF CM cultures, this was not statistically different due to the extent of variance between replicates (Figure 6). In these cultures, GF CM showed minimal difference compared to normal media cultures with the latter exhibiting less variance and thus a significantly lower ALP activity than PDL CM culture. Compared to the other GF cultures, GF4 had much lower levels of ALP activities when cultured in normal media and GF CM. GF4 cells cultured in PDL CM had significantly higher ALP activity compared to those in GF CM and normal media. Overall, these results exhibited a similar pattern with the ALP gene expression in the previous experiments (Figure 5D).

### 3.4 Effect of Dkk‐1 on ALP activity in conditioned medium‐treated cells

**FIGURE 7:**
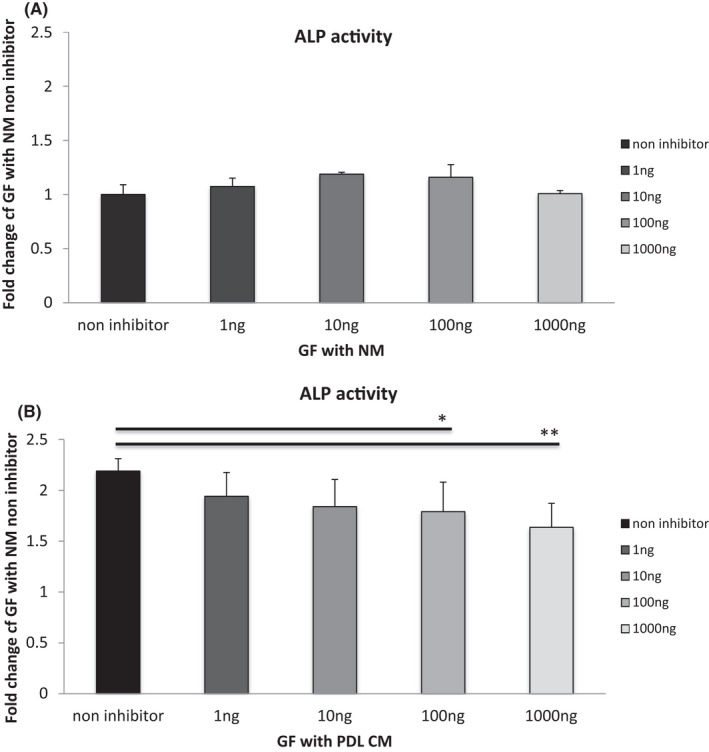
Effect of Wnt inhibition on alkaline phosphatase (ALP) activity in gingival fibroblasts (GFs) cultured with PDL conditioned medium (PDL CM). GFs were cultured in PDL CM and normal media supplemented with the Wnt inhibitor, Dkk‐1. Non‐inhibitor samples were used as control. Fold change was calculated relative to non‐inhibitor samples in normal media. Data represented as mean ± *SD*. *n* = 3 replicates, and the experiment was repeated twice. One‐way ANOVA with Bonferroni's post‐test was used for statistical analysis. * *p* < .05; ** *p* < .01; ***p < .001. Only statistically significant differences indicated

Dkk‐1 had no effect on ALP activity in cells grown in normal medium (Figure 7a). In contrast, addition of Dkk‐1 to GF cultures treated with PDL CM resulted in a dose‐dependent inhibition of the effect of PDL CM on ALP activity (Figure 7b). In addition, in further experiments with GF cell lines GF‐1 and GF‐2, PDL CM induced ALP activity, and either addition of 100 ng/ml or 1 µg/ml Dkk‐1 significantly decreased the effect of the PDL CM.

## 4 DISCUSSION

We postulated that periodontal ligament–derived MSCs could induce expression of periodontal ligament markers in gingival cell cultures. Our results consistently demonstrate that PDL cells could induce expression of the PDL‐associated genes asporin and periostin together with alkaline phosphatase, and, to a lesser extent, nestin expression in GF cultures. The results demonstrate that this effect was seen in a range of GF lines, with different PDL lines. Using a range of co‐cultures and conditioned medium, the results suggest that the effect was the result of secretion of diffusible/soluble signaling molecules, as the effects were evident in transwell cultures where cell types are physically separated and in the conditioned medium of PDL cells, as well as in direct co‐cultures.

Interestingly, GF appeared to have the opposite effects on PDL cells resulting in down‐regulation of asporin and periostin in PDL cells. Both asporin and periostin are clearly compartmentalized to the PDL rather than gingival connective tissue anatomically.^19^ Although this was not a primary aim of the studies here, the inhibitory effect of GF on PDL cells is consistent with previous findings in our laboratory demonstrating the inhibition of osteogenesis by GF through secretion of the BMP inhibitor, Gremlin‐1.^20^ These findings suggest that there is bi‐directional paracrine signaling between GF and PDL cells that may play an important functional role in periodontal tissue homeostasis, as well as during tissue repair following damage. This is of particular relevance in the dentogingival junction where gingival connective tissue and periodontal ligament directly approximate. Clinical techniques such as guided tissue regeneration exclude the gingival connective tissue from underlying the periodontal apparatus to prevent encroachment by gingival cells and thus preserve wound space, but inhibitory signaling from the gingival cells might also be avoided. This could be potentially targeted to produce novel treatment strategies to produce favorable wound healing outcomes in periodontal regeneration therapies.

MSCs have been shown to have a complex secretome which appears to make a major contribution to the properties of MSC such as immunomodulation, homing, cell recruitment, and differentiation.^21^, ^22^ There is now considerable interest in the potential therapeutic application of MSC secreted products and the possible exploitation of MSC properties in cell‐free applications. Furthermore, MSCs have been shown to produce exosomes, which are vesicles released by cells, which contain a complex mix of growth factors and cytokines, other signaling molecules, and miRNAs.^23^ The possible use of exosomes and other MSC‐derived preparations is now being actively researched for treatment in a wide range of conditions, including induction of periodontal regeneration.^24^ To date, the potential application of PDL MSC‐derived factors to regulate periodontal regeneration has shown modest potential in early preclinical models^25^, ^26^ and suggests recruitment of endogenous host cells by the applied factors. However, factor isolation protocols are highly variable, and characterization of factors and mechanisms of their actions are largely unknown. In preliminary experiments, here we demonstrated that the effect on ALP expression and alkaline phosphatase protein production was blocked by addition of the Wnt signaling inhibitor Dkk1, implicating Wnt signaling as a potential pathway for this interaction. Interestingly, our group has also previously found another Wnt signaling inhibitor Sfrp4 expressed at higher levels in PDL cells than in GF‐4.^19^ Given the potential complexity of the PDL secretome, including that from exosomes, it would not be surprising if additional multiple pathways were involved in these signaling processes and further investigation of this would be useful in the future.

One of the most striking findings of the studies here is the extensive heterogeneity observed between different GF lines. A number of previous studies have demonstrated the presence of MSC populations in GF cultures derived from both healthy and inflamed gingival tissues.^12^, ^13^ These cells have been shown to exhibit the characteristics of mesenchymal stem cells as set out by the International Society of Cellular Therapy (ISCT) including expression of cell surface markers such as CD34, CD90, CD105, and CD146, are clonogenic, and have multilineage differentiation potentials. In our studies, here we used 4 different cell lines isolated from different donors. All of these 4 lines contained populations of cells which expressed these MSC‐associated cell surface markers to a significant extent and showed multilineage differentiation capacities and expression of PDL‐associated marker genes. However, the proportions of MSC‐like cells varied significantly between cell lines. In particular, one of the cell lines, GF‐4, had a markedly lower proportion of cells expressing MSC cell surface markers and also showed low basal levels of expression of PDL markers. This gingival cell line had been commercially obtained, and unfortunately, there was no additional information provided as to the isolation method beyond the fact that it was an explant culture from a gingival tissue sample. The other 3 lines we used were isolated in our laboratory by explant culture from the collar of gingival tissue removed with tooth extraction, meaning that the anatomical derivation of these cells is largely from the region of the dentogingival junction.

We have recently shown that there is a clear distinction between periodontal and gingival tissue phenotypes at the dentogingival junction^19^ and it is thus possible that our gingival explant cultures represent a variable mixture of gingival and PDL cell phenotypes which would explain the marked heterogeneity between cultures. These findings further emphasize the general point of the marked heterogeneity of primary gingival fibroblast culture phenotypes.

## 5 CONCLUSION

Our findings reveal that PDL cells secrete diffusible factors which can induce the expression of PDL markers in gingival fibroblast cultures. These findings emphasize the potential for putative PDL stem cells to contribute to tissue regeneration by recruitment of host cells and further suggest the possibility that this activity could be harnessed therapeutically without the need for exogenous stem cell implantation. Additional studies are required to investigate further the mechanisms of this activity and the specific subpopulations of cells which may be targets for this activity.

## Supporting information

Fig S1‐S5

Supplementary Material

Supplementary Material
