# Supplementary material for: Regulation of gingival fibroblast phenotype by periodontal ligament cells in vitro
Source: J Periodontal Res. 2022 Jan 17;57(2):402–11. doi: 10.1111/jre.12971 (PMC9302626; doi:10.1111/jre.12971)
Supplement: Supplementary file 1 — Fig S1‐S5 [file JRE-57-402-s003.pptx]

## Slide 1
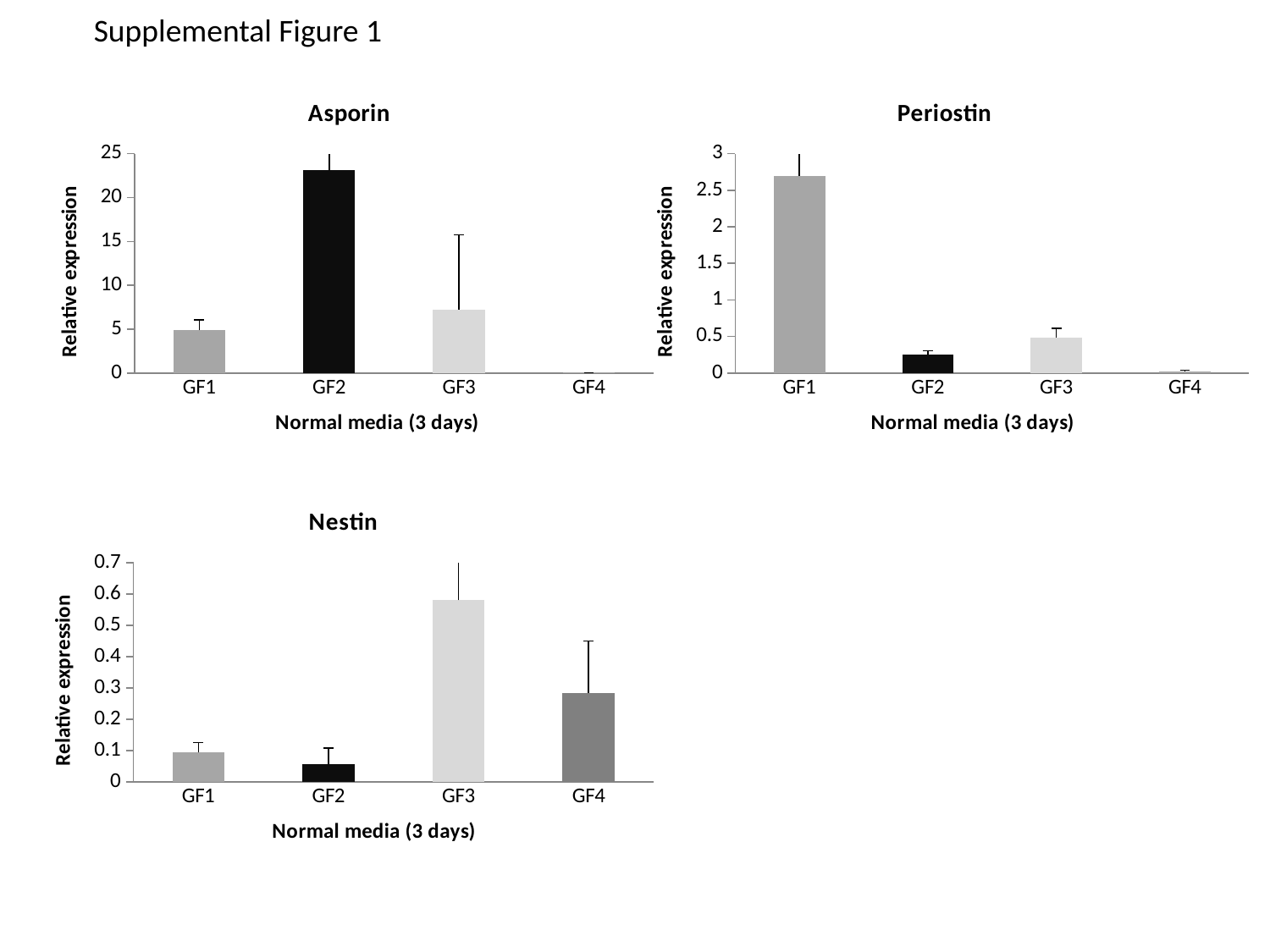

Supplemental Figure 1
### Chart: Asporin
| Category | |
|---|---|
| GF1 | 4.871764740737706 |
| GF2 | 23.09397270390528 |
| GF3 | 7.220750963307371 |
| GF4 | 0.0461814309161887 |
### Chart: Periostin
| Category | |
|---|---|
| GF1 | 2.697707511133433 |
| GF2 | 0.255814049303681 |
| GF3 | 0.485531655486666 |
| GF4 | 0.0268678577662684 |
### Chart: Nestin
| Category | |
|---|---|
| GF1 | 0.0937680514954861 |
| GF2 | 0.0579470817666203 |
| GF3 | 0.580059647171059 |
| GF4 | 0.284555875897264 |

## Slide 2
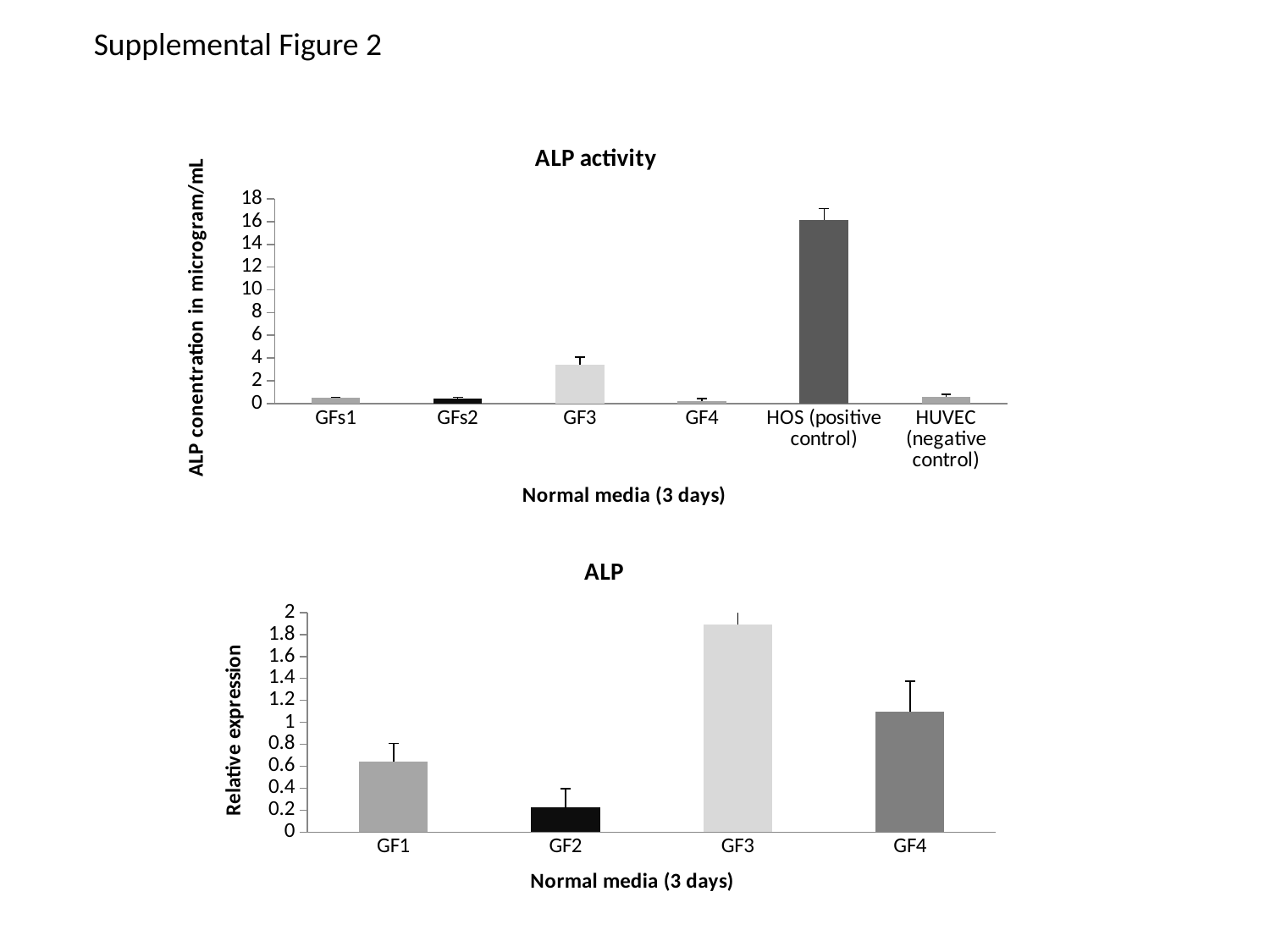

Supplemental Figure 2
### Chart: ALP activity
| Category | |
|---|---|
| GFs1 | 0.53528773072747 |
| GFs2 | 0.45928338762215 |
| GF3 | 3.380342 |
| GF4 | 0.21794855 |
| HOS (positive control) | 16.13787875 |
| HUVEC (negative control) | 0.5598291 |
### Chart: ALP
| Category | |
|---|---|
| GF1 | 0.641407148141497 |
| GF2 | 0.2267904390802 |
| GF3 | 1.88883622144635 |
| GF4 | 1.100385738528562 |

## Slide 3
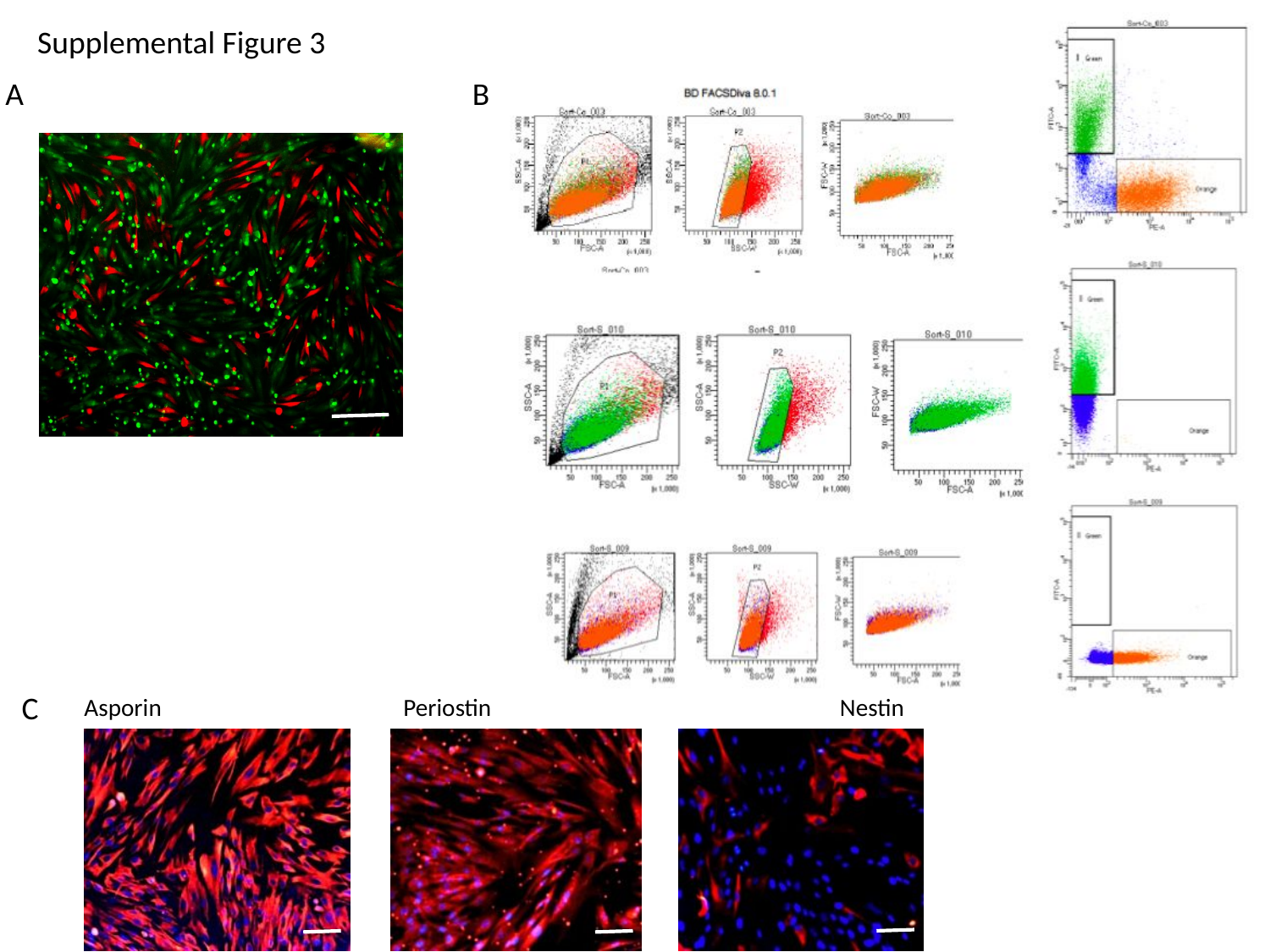

Supplemental Figure 3
A
B
C
 Asporin	 Periostin	 	 Nestin

## Slide 4
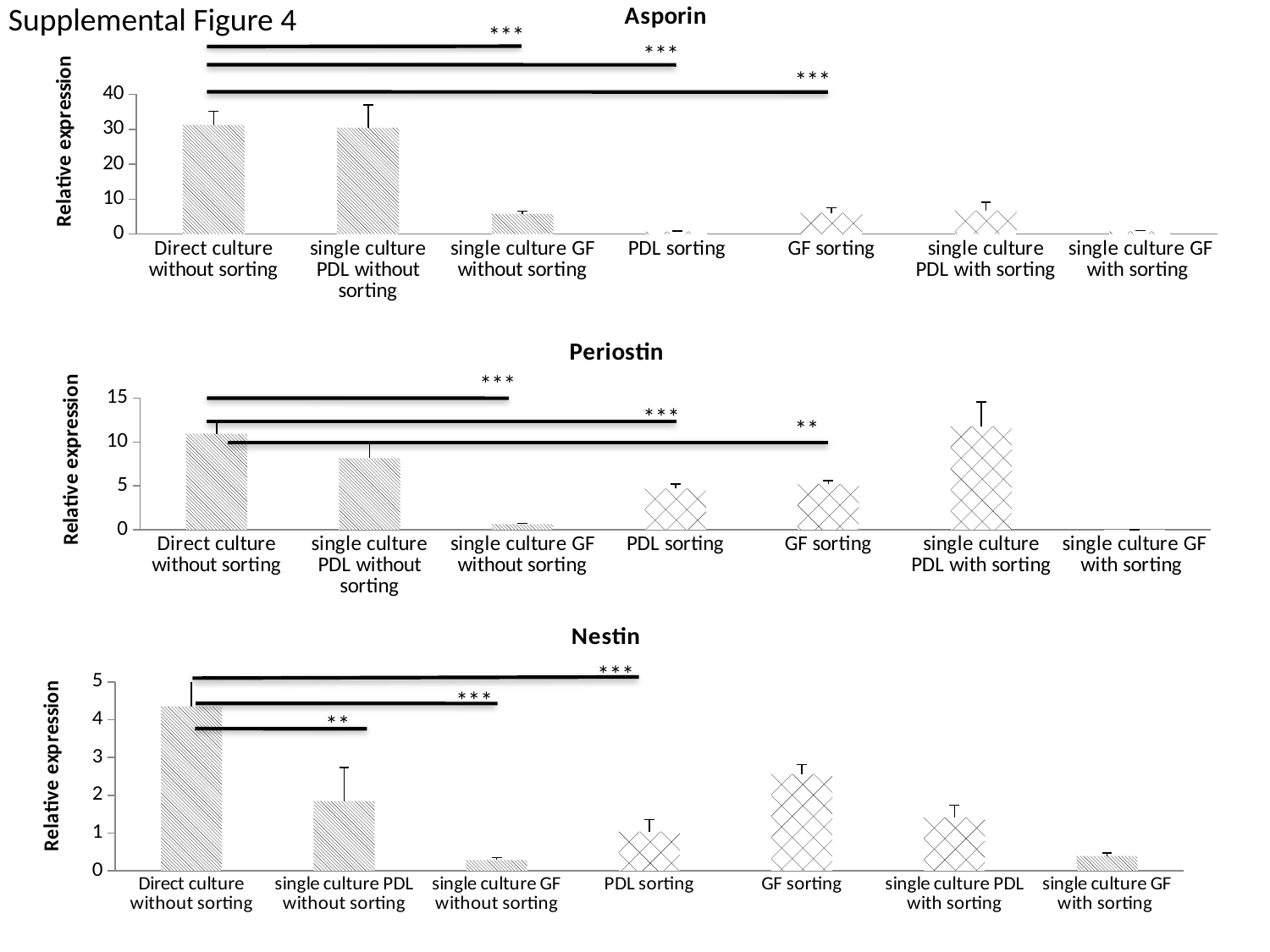

### Chart: Asporin
| Category | |
|---|---|
| Direct culture without sorting | 31.22910528453552 |
| single culture PDL without sorting | 30.46384888709541 |
| single culture GF without sorting | 5.770219296601044 |
| PDL sorting | 0.685230317731418 |
| GF sorting | 6.057625242043486 |
| single culture PDL with sorting | 6.677826288838871 |
| single culture GF with sorting | 0.73984113486473 |Supplemental Figure 4
### Chart: Periostin
| Category | |
|---|---|
| Direct culture without sorting | 10.94277312524224 |
| single culture PDL without sorting | 8.15703301274882 |
| single culture GF without sorting | 0.628984186686254 |
| PDL sorting | 4.698015671112635 |
| GF sorting | 5.207319060113272 |
| single culture PDL with sorting | 11.77254984971717 |
| single culture GF with sorting | 0.0175571100045217 |***
**
### Chart: Nestin
| Category | |
|---|---|
| Direct culture without sorting | 4.349425659505829 |
| single culture PDL without sorting | 1.846660498685281 |
| single culture GF without sorting | 0.28460959465592 |
| PDL sorting | 1.039024264936419 |
| GF sorting | 2.560961691660726 |
| single culture PDL with sorting | 1.406973327628636 |
| single culture GF with sorting | 0.391909967950214 |***
***
**

## Slide 5
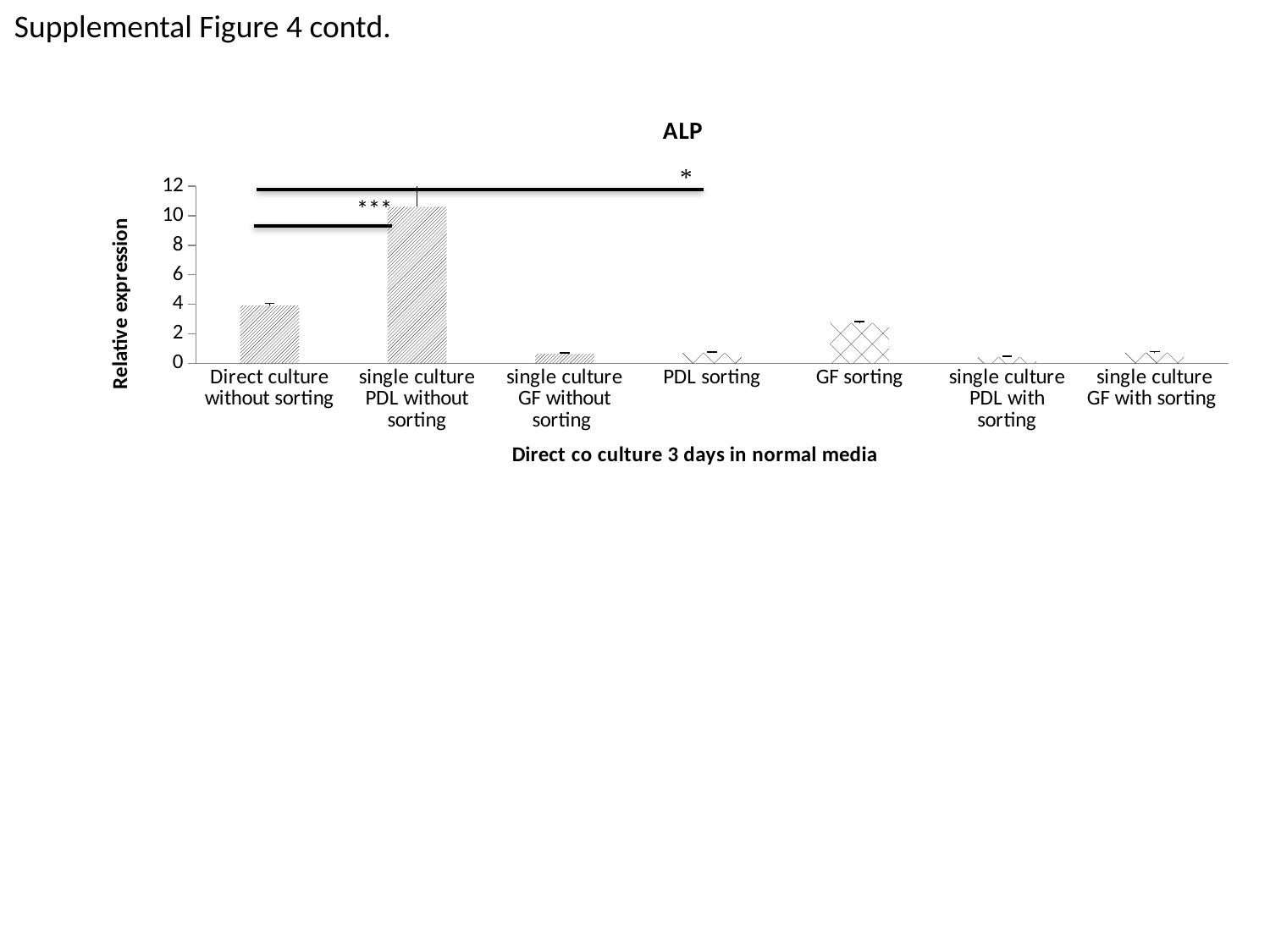

Supplemental Figure 4 contd.
### Chart: ALP
| Category | |
|---|---|
| Direct culture without sorting | 3.92479582467317 |
| single culture PDL without sorting | 10.5994219846606 |
| single culture GF without sorting | 0.641407148141497 |
| PDL sorting | 0.701398332451569 |
| GF sorting | 2.726266474259336 |
| single culture PDL with sorting | 0.419471626545127 |
| single culture GF with sorting | 0.719716053967862 |

## Slide 6
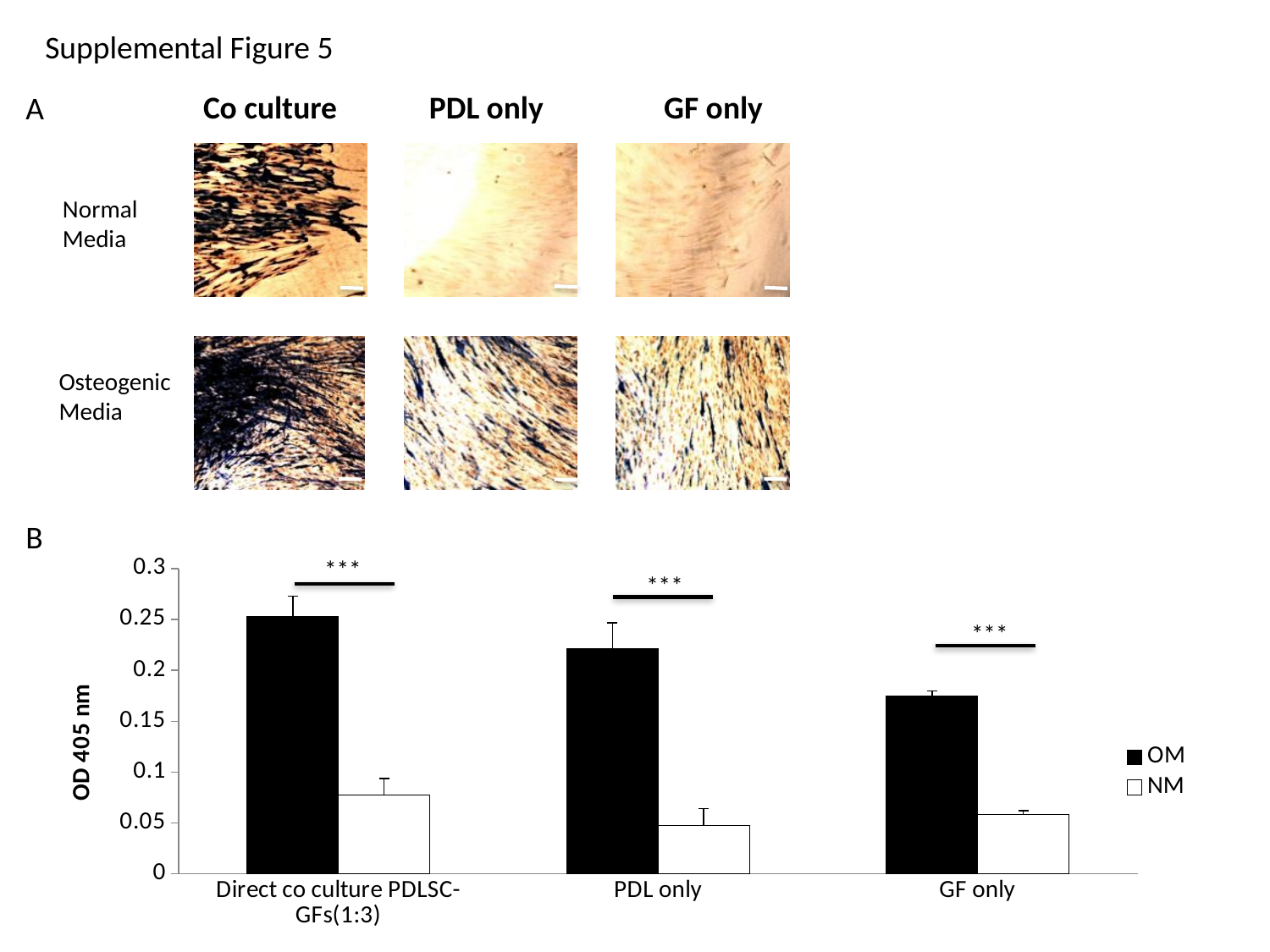

Supplemental Figure 5
A
| Co culture | PDL only | GF only |
| --- | --- | --- |
| | | |
| | | |
Normal Media
Osteogenic Media
### Chart
| Category | OM | NM |
|---|---|---|
| Direct co culture PDLSC-GFs(1:3) | 0.253166666666667 | 0.0775 |
| PDL only | 0.2215 | 0.0476666666666667 |
| GF only | 0.17475 | 0.0585 |***
***
***
B
