# Supplementary material for: Regulation of gingival fibroblast phenotype by periodontal ligament cells in vitro
Source: J Periodontal Res. 2022 Jan 17;57(2):402–11. doi: 10.1111/jre.12971 (PMC9302626; doi:10.1111/jre.12971)
Supplement: Supplementary file 2 — Supplementary Material [file JRE-57-402-s002.docx]

Supplemental Figure Legends

**Supplemental Figure 1 Periodontal marker gene expression level in 4 primary gingival fibroblast (GF) lines**

GFs were cultured in normal media for 3 days. Marker expression levels were assessed by qPCR and expression normalised to RPL13a expression. Data represented as mean ± S.D. Three replicates were tested for each cell line. Two-way ANOVA with Bonferroni’s post-test was used for statistical analysis. (F=20.11 p<0.001).

**Supplemental Figure 2 Alkaline phosphatase gene expression and activity in 4 primary gingival fibroblast (GF) lines**

GFs were cultured in osteogenic media for 3 days. Alkaline phosphatase activity was determined by colormetric p-nitrophenol assay. Marker expression levels were assessed by qPCR and expression normalised to RPL13a expression. Data represented as mean ± S.D. Three replicates were tested for each cell line. Human osteosarcoma cells (HOS) and HUVEC were used for positive and negative control respectively. Error bars indicated standard and one-way ANOVA with Bonferroni post hoc was used for statistical analysis (*, p<0.05; **. p<0.01 and ***, p<0.001).

**Supplemental Figure 3 Cell sorting of co-cultures with fluorescence activated cell sorting (FACS).**

Periodontal ligament cells (PD) (n=3) and gingival fibroblasts (GF) (n=1) were stained with cell tracker orange and cell tracker green respectively (A). Both cells were co-cultured directly in the ratio of 1:3, grown in normal media for 3 days. Based on cell labelling, FACS separated PDL and GF direct co-cultured labelled cells (B) (upper row), GF only (middle row), and PDL only (bottom row) were also sorted the same way as direct co culture. (C) Immunocytochemical staining for asporin (left), periostin (middle) and nestin (right) in PDL/GF direct co-cultures. Scale bar – 100um.

**Supplemental Figure 4 Periodontal marker expression in direct co-cultured periodontal cells.**

Periodontal ligament cells (PDL) (n=3) and gingival fibroblasts (GFs) were co cultured directly in a PDL:GF ratio of 1:3. All cells were seeded in triplicates and grown in normal media for 3 days. Co-cultures were divided into two groups, without sorting and following sorting. Representative data of three independent experiments shown. Data presented as mean ± S.D. One-way ANOVA with Bonferroni’s multiple comparisons was used for statistical analysis. (*, p<0.05; **, p<0.01; ***, p<0.001). All the asterisks showed the comparison with direct co-culture without sorting.

Supplemental Figure 5 Histochemical determination of alkaline phosphatase (ALP) staining in periodontal cells

A. Light micrograph showing Fast Blue BB staining of ALP in direct co-cultured periodontal ligament cells (PDLs) and gingival fibroblasts (GFs) with the ratio of 1:3 in normal media. Scale bar – 50um. B. Spectrophotometric intensity of wells in A. (n=3) , ***, p<0.0001, one way ANOVA with Bonferroni post-test).
