# Supplementary material for: Regulation of gingival fibroblast phenotype by periodontal ligament cells in vitro
Source: J Periodontal Res. 2022 Jan 17;57(2):402–11. doi: 10.1111/jre.12971 (PMC9302626; doi:10.1111/jre.12971)
Supplement: Supplementary file 3 — Supplementary Material [file JRE-57-402-s001.docx]

**Supplemental Methods**

**Osteogenic differentiation**

PDLSCs were seeded at a density of 1.2x10^4^ cell/cm^2^ into 12-well and 24-well plates (Nunc, Fisher Scientific, Loughborough, UK) and after 24 hours were treated with osteogenic medium consisting of growth medium supplemented with 0.1 μM dexamethasone, 0.05 mM ascorbic acid and 10 mM ß-glycerophosphate (Sigma–Aldrich). The osteogenic inductive medium was replaced twice a week. Osteogenic differentiation was assessed by the accumulation of calcium deposits by staining with alizarin red dye. Cells were fixed within 15 min. with 10% neutral buffered formalin, stained for 5 minutes with alizarin red S with 1:100 dilution in water and washed five times in 50% ethanol and air-dried. Mineralized deposits of calcium stained a red colour. After being stained, the cells were dried at room temperature.

####

#### Adipogenic differentiation

To induce adipogenic differentiation cells were again seeded at a density of 4 x 10^4^ cell/cm^2^ into 12-well and 24-well plates incubated for 24 hours before changing to adipogenic medium containing growth medium supplemented with 1 μM dexamethasone, 0.25 mM isobutylmethylxanthine, 50 μM indomethacin and 10 μg/ml insulin (Sigma–Aldrich). The adipogenic inductive medium was replaced twice a week and results evaluated at 3 days, 7 days, 14 days, 21 days and 28 days.

Detecting lipid nodule stained with Oil-Red-O dye assessed adipogenesis. Cells were fixed within 15 minutes with 10% neutral buffered formalin, stained for 15 minutes with Oil-Red-O and washed once with 60% isopropanol and 2 times with PBS. Lipid vacuoles within adipocytes stained a red color could be detected under microscope.

####

#### Chondrogenic differentiation

Cells were seeded at a density of 2.5 x 10^5^ cell/cm^2^ in 96-well, 12- well and 24-well plates (Nunc) with chondrogenic inductive medium containing of αMEM supplemented with 10% FBS, 1 μM dexamethasone, 1 μM ascorbic acid, 1% sodium pyruvate (Invitrogen) and 10ng/ml transforming growth factor-beta 1 (TGF-β1, Peptrotech, Rocky Hill, NJ, USA). For 96-well and 24-well plates, plates were spun in a bench top centrifuge at 500x g for 5 minutes. Twenty-four hours after centrifugation, the aggregate were allowed to float freely by releasing them from the bottom of the wells by aspirating 100μl of media and gently releasing it back into the wells. The chondrogenic inductive medium was changed two times a week.

Detecting cartilage matrix molecules stained with Alcian Blue assessed chondrogenesis. Cells were fixed within one hour with 10% neutral buffered formalin, stained with Alcian Blue and incubated for overnight at room temperature in the dark and washed two times for 20 minutes with destaining solution containing 120ml 98-100% Ethanol with 80 ml 98-100% Acetic Acid. Cartilage matrix molecules namely glycosaminoglycans would stain intense dark-blue, whereas other tissue was at most faintly bluish.

**Flow cytometry**

MSC surface marker expression was measured using four-colour flow cytometry analysis, with the FACSCanto II flow cytometer (BD Biosciences, San Jose, CA).

Briefly, 100,000 cells were incubated with Fc block (BioLegend, Cambridge, U.K.) in 50μl of fluorescence-activated cell sorting (FACS) buffer for 15 minutes at room temperature. Antibody cocktail was divided into two groups (A&B). Group A contained CD34-PerCP/Cy5.5 (clone 4H11), CD105-fluorescein isothiocyanate (FITC) (clone 43A3), CD146-phycoerythrin, and (PE)/Cy7 (clone SHM-57) whereas group B contained CD45-PerCP/Cy5.5 (clone HI30), CD44-FITC (clone BJ18), and CD90-allophycocyanin (APC) (clone 5E10). Adding an antibody cocktail into the FACS tube, which contained the cells suspension, promoted direct binding.

Unstained cells, compensations beads, and fluorescence minus one (FMO) were used to set the gating. All antibodies were obtained from Bio-Legend U.K. (Cambridge Bioscience) and used at the recommended concentration. Cells were incubated for 15 minutes at room temperature in the dark. After being washed twice, cells were re suspended in 300μl of FACS buffer and immediately being analysed. A total of 10,000 events were acquired for each sample and data analysis was performed using DIVA software (BD Biosciences).
